# Drought and Root Herbivory Interact to Alter the Response of Above-Ground Parasitoids to Aphid Infested Plants and Associated Plant Volatile Signals

**DOI:** 10.1371/journal.pone.0069013

**Published:** 2013-07-19

**Authors:** Muhammad Tariq, Denis J. Wright, Toby J. A. Bruce, Joanna T. Staley

**Affiliations:** 1 Division of Biology, Faculty of Natural Sciences, Imperial College London, Silwood Park Campus, Ascot, United Kingdom; 2 Department of Biological Chemistry, Rothamsted Research, Harpenden, United Kingdom; 3 NERC Centre for Ecology and Hydrology, Maclean Building, Crowmarsh Gifford, Wallingford, United Kingdom; INRA-UPMC, France

## Abstract

Multitrophic interactions are likely to be altered by climate change but there is little empirical evidence relating the responses of herbivores and parasitoids to abiotic factors. Here we investigated the effects of drought on an above/below-ground system comprising a generalist and a specialist aphid species (foliar herbivores), their parasitoids, and a dipteran species (root herbivore).We tested the hypotheses that: (1) high levels of drought stress and below-ground herbivory interact to reduce the performance of parasitoids developing in aphids; (2) drought stress and root herbivory change the profile of volatile organic chemicals (VOCs) emitted by the host plant; (3) parasitoids avoid ovipositing in aphids feeding on plants under drought stress and root herbivory. We examined the effect of drought, with and without root herbivory, on the olfactory response of parasitoids (preference), plant volatile emissions, parasitism success (performance), and the effect of drought on root herbivory. Under drought, percentage parasitism of aphids was reduced by about 40–55% compared with well watered plants. There was a significant interaction between drought and root herbivory on the efficacy of the two parasitoid species, drought stress partially reversing the negative effect of root herbivory on percent parasitism. In the absence of drought, root herbivory significantly reduced the performance (e.g. fecundity) of both parasitoid species developing in foliar herbivores. Plant emissions of VOCs were reduced by drought and root herbivores, and in olfactometer experiments parasitoids preferred the odour from well-watered plants compared with other treatments. The present work demonstrates that drought stress can change the outcome of interactions between herbivores feeding above- and below-ground and their parasitoids, mediated by changes in the chemical signals from plants to parasitoids. This provides a new insight into how the structure of terrestrial communities may be affected by drought.

## Introduction

Plants, insect herbivores and the natural enemies (predators and parasitoids) of insects interact in multitrophic food webs that influence community dynamics [1]–[4]. Plants are simultaneously challenged by above- and below-ground insect herbivores that can affect one another through plant-mediated interactions [5]–[8]. Below-ground herbivores can increase water stress in plants [9], induce changes in plant physiology that are similar to drought [5], and may strongly affect the quality and quantity of nutrients and secondary metabolites available to other herbivores [10], [11]. Such physiological and chemical changes produce a variety of responses within plants that can directly influence foliar insects [11]–[14] and their predators and parasitoids [14]. The impact of root herbivores on the performance of foliar herbivores can be positive [5], [11], [13], [15], [16], negative [17]–[19] or neutral [20], depending on the mechanism by which they interact, and the order of arrival on a host plant [21], [22].

Drought stress may affect herbivorous insect performance, diversity and abundance indirectly via changes in plant physiology [23]–[27]. Increases in the frequency, duration, and/or severity of drought are predicted in several geographic regions under current climate change models [28] and can alter the structure and composition of terrestrial ecosystems [29]. Under current climate change predictions, much of the globe would experience dryness on a far greater scale and frequency than those that assessed previously [30]. With a medium increase in CO_2_ emissions, the levels of soil moisture are likely to decrease and low soil moisture is increasingly regarded as a potential contributor to heat waves and drought [31]. Despite these large changes in rainfall under climate change, few studies have empirically addressed the effects of climate change factors on multitrophic interactions [32], [33].

It has been suggested that insect herbivore performance and populations increase on drought stressed plants due to an increase in the availability of nutrients [24], [34] and/or a decrease in the concentration of defensive compounds [35]. However, more recent studies have shown that drought stress can have both positive and negative effect on foliar herbivores depending on stress intensity [36] and herbivore feeding guild [37]. Research has highlighted the complexity of aphid plant interactions under drought stress, where it has been observed that the high drought stress had negative impact on aphid performance [36]–[39] even though the drought stressed plants had higher concentrations of nitrogen [27], [36] and amino acids [38]. In contrast, under more moderate levels of drought stress aphid performance and populations may increase [36]. The effects of drought stress on herbivores are well documented [23], [36], [38], [40] but the indirect effects on parasitoids are less well understood [41]. Only a few studies have demonstrated that drought stress has a negative effect on aphid parasitism success [39], [42].

Root herbivores can affect plant growth [43]–[45], reproduction [46], [47], density [46], [48] and nutrient status [49], [50] and thus may strongly affect the quality and quantity of resources available to foliar herbivores [36]. This can have differential effects on foliar insects and their associated natural enemies. Root herbivory had a negative impact on the performance of aphids and other insects due to the increased levels of defence compounds in several studies [6], [19], [36], [51]–[57] and/or a decrease in nitrogen concentration [19] and leaf water content [58]. Root herbivores can be responsible for the change in growth and development of foliar herbivores through plant mediated changes and thus may have indirect impact on parasitoid fitness [19], [59], [60] and the impact can also be seen on the fourth trophic level [19]. The negative impact of high drought stress on aphid performance and abundance can be exacerbated under root herbivory [36], [39], [61] and thus we predict that natural enemies may avoid these plants due to the low quality of their aphid hosts.

Multitrophic interactions frequently involve complex plant defences [10], [14], [62] involving the release of volatile organic compounds (VOCs) following herbivore attack that enhance the effectiveness of natural enemies [63]–[67]. In response to insect herbivory, plants release VOCs which can be used by natural enemies of the insect herbivores to find their hosts [59]. The plant VOC emissions induced by foliar herbivores can be influenced by root herbivores [59] and drought stress [68]. These studies showed compound specific responses for natural enemies under biotic and abiotic stresses. Therefore, plant VOC emissions are influenced by biotic and abiotic stresses [68]–[73]. These plants may become less attractive to foraging parasitoids [74], [75] and thus may interfere directly with herbivore-parasitoid interactions [59].

The behaviour and performance of natural enemies can be influenced by their host, host diet, environmental factors (including water stress) and the presence of other herbivores such as root feeders [26], [42], [59], [79]–[81]. Parasitoid development has been linked with the quality of internal environment of their hosts [59]. For example, phytotoxin concentration can increase under drought stress [25] and root herbivory [59] and these toxins are repeatedly consumed by insect herbivores [59]. These phytotoxins often accumulate in the fat body and hemolymph of insect herbivores which may have a negative impact on the fitness of developing parasitoid larvae [59]. Parasitoids may thus be particularly sensitive to changes in their prey diet and environmental conditions [82], [83].

Some studies have examined the effects of above- or below-ground interactions in multitrophic systems [39], [59], [74], [76], [77], but there have been very few studies on the effects of abiotic factors on both above- and below-ground interactions [61], [78], and none comparing the response of two parasitoid species to below-ground herbivory in conjunction with abiotic stress. In the last decade, studies have linked multitrophic (above-below ground) interactions with either to observe the impact on parasitoid performance or changes in plant VOCs, but few addressed both aspects together [59]. The main objective of the present study was to examine how a multitrophic system with above- and below-ground components was influenced by drought stress. The second objective was to examine how root herbivory and drought stress affects above-ground host parasitoid interactions, potentially mediated by changes in plant VOC emissions.

We hypothesised that: (1) high levels of drought stress and below-ground herbivory interact to reduce the performance of parasitoids developing in aphids; (2) drought stress and root herbivory change the profile of volatile organic chemicals (VOCs) emitted by the host plant; (3) parasitoids avoid aphid hosts feeding on plants under drought stress and root herbivory. The system comprised *Brassica oleracea* as the host plant; the belowground herbivore was the cabbage root fly *Delia radicum*; the aboveground herbivores were the generalist aphid *Myzus persicae*, and the specialist aphid *Brevicoryne brassicae*; and at the third trophic level the parasitoids *Aphidius colemani* and *Diaeretialla rapae* were used.

## Results

### Parasitism Performance and Percentage Parasitism

**Figure 1:**
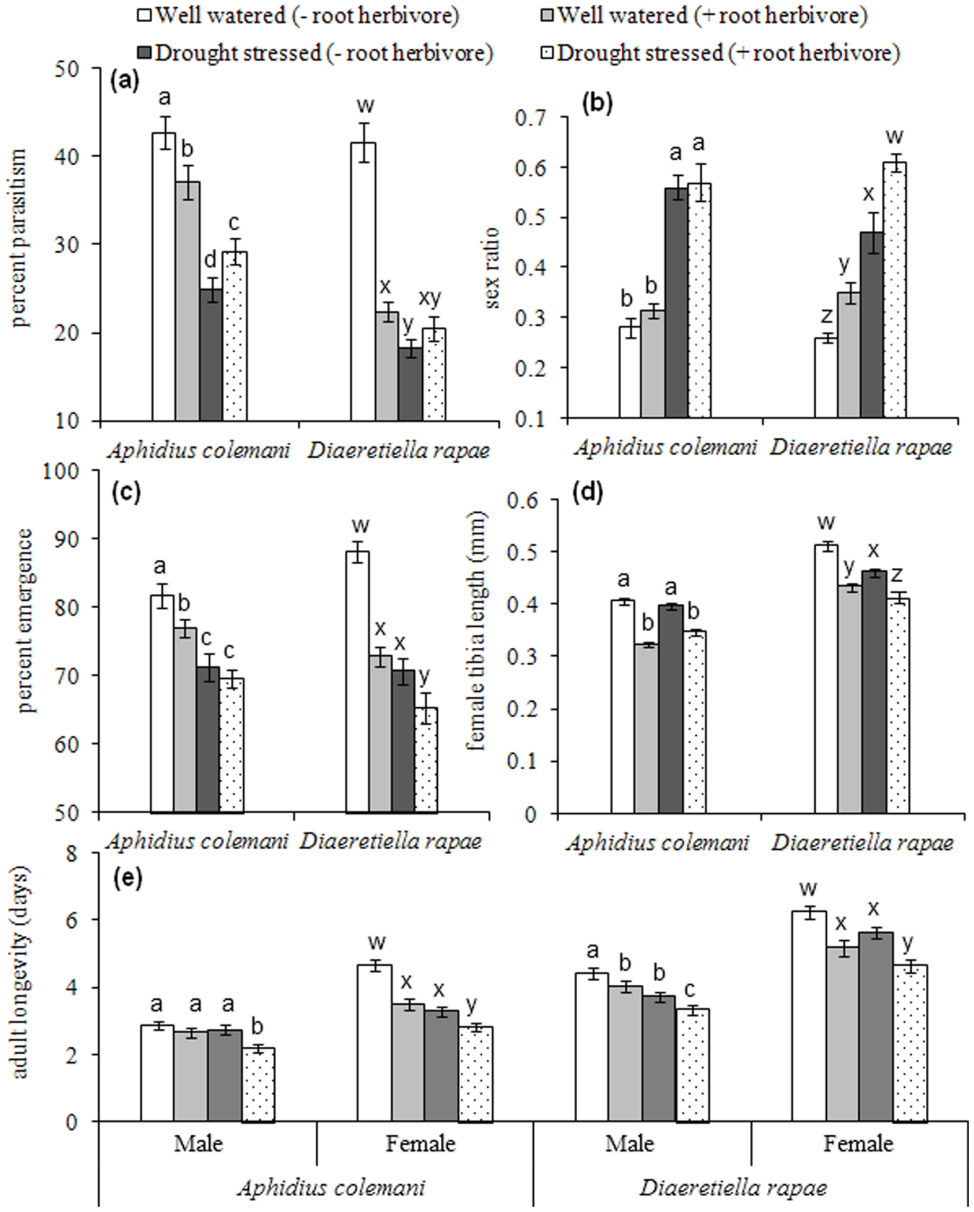
Performance of *Aphidius colemani* and *Diaeretiella rapae* (mean ± S.E.M.) of *Myzus persicae* and *Brevicoryne brassicae* reared on *Brassica oleracea* plants under a well-watered regime (200 ml/pot/week; “Control”) and a reduced water regime (100 ml/pot/week; “Drought stressed”) with/without *Delia radicum*. Within each parasitoid species, means with different letters are significantly different (P<0.05): (a) Percentage parasitism (b) sex ratio (c) percentage emergence (d) female tibia length (mm) and (e) adult longevity (days). A high sex ratio indicates a high proportion of male parasitoids.

- **Percentage parasitism.** Percentage parasitism was significantly affected by the interaction between drought stress, *De*. *radicum* and parasitoid species (*F*_1, 72_ = 7.50; *P*<0.01). Drought stress (*F*_1, 72_ = 121.39; *P*<0.001) and the presence of *De*. *radicum* (*F*_1, 72_ = 10.27; *P*<0.01) had a negative impact on percentage parasitism by both parasitoid species compared with well watered plants, but their effects were greater for the specialist parasitoid species (*D*. *rapae*) than for the generalist parasitoid species (*A*. *colemani*, Figure 1a). Drought stress partially reversed the negative effect of *De*. *radicum* on parasitism by *A*. *colemani* (Figure 1a; Tukey’s HSD, *P*<0.05). Parasitism by *D. rapae* followed the same pattern, but the difference between drought stressed plants with or without *De*. *radicum* was not significant (Figure 1a).
- **Sex ratio.** Sex ratio was significantly affected by the interaction between *De*. *radicum* treatment and parasitoid species (*F*_1, 75_ = 7.35; *P*<0.01). The main effects of drought stress (*F*_1, 75_ = 19.65; *P*<0.001) and *De*. *radicum* (*F*_1, 75_ = 215.93; *P*<0.001) were also significant for the sex ratio of both parasitoid species. The proportion of males of both species was significantly greater on drought stressed plants with *De*. *radicum* compared with well watered treatments (Figure 1b). *Delia radicum* increased the proportion of male *D*. *rapae* on both drought stressed plants and well watered plants compared with plants that were not infested with root herbivore (Tukey’s HSD, *P*<0.05). *Delia radicum* feeding did not affect the sex ratio of *A*. *colemani* under either the drought or well watered treatments (Tukey’s HSD, *P*<0.05).
- **Percentage emergence.** Percentage emergence of adult parasitoids was significantly affected by the interactions between drought stress and *De*. *radicum* treatments (*F*_1, 74_ = 6.81; *P*<0.05) and parasitoid species and *De*. *radicum* treatments (*F*_1, 74_ = 8.11; *P*<0.01). Emergence was maximised on well watered plants without *De*. *radicum* for both parasitoid species, and minimised under the combined drought stress and *De*. *radicum* treatments (Tukey’s HSD, *P*<0.05; Figure 1c).
- **Female tibia length.** Female tibia length was significantly affected by interactions between drought stress x *De*. *radicum* treatment (*F*_1, 74_ = 9.24; *P*<0.01) and drought stress x parasitoid species (*F*_1, 74_ = 11.58; *P*<0.01). The tibia length of both parasitoid species decreased significantly in the *De*. *radicum* treatment under both the drought stress and the well watered treatment (Tukey’s HSD, *P*<0.05; Figure 1d). The mean tibia length of *A. colemanii* was unaffected by drought stress, whereas that of *D. rapae* was slightly reduced under drought stress, but more affected by the *De*. *radicum* treatment (Tukey’s HSD, *P*<0.05).
- **Adult longevity.** The interaction between drought stress, *De*. *radicum* and parasitoid species was significant (*F*_1, 72_ = 4.58; *P*<0.05). Female adult longevity was significantly affected by drought stress (*F*_1, 72_ = 29.77; *P*<0.001) and *De*. *radicum* (*F*_1, 72_ = 29.73; *P*<0.001), which was maximised for both parasitoid species on well watered plants without *De*. *radicum* and minimised on plants with both drought stress and *De*. *radicum* treatment (Figure 1e; Tukey’s HSD, *P*<0.05). Drought stress (*F*_1, 76_ = 18.90; *P*<0.001) and *De*. *radicum* (*F*_1, 76_ = 16.88; *P*<0.001) had a significant effect on adult male longevity for both parasitoid species (*F*_1, 76_ = 122.05; *P*<0.001). Males of both parasitoid species had shorter adult longevity compared with females (Figure 1e).

### Parasitoid Response to Plant Volatiles (Olfactometer Experiment)

Both parasitoid species preferred the well watered plants compared to plants either under drought stress, root herbivory or both (Table 1). Parasitoid preference decreased significantly with drought stress. Preference of *D*. *rapae* was similar on drought stressed plants with or without *De*. *radicum* root herbivory. However, *A. colemani* preferred drought stressed plants without *De. radicum* to drought stressed plants with *De. radicum*. Furthermore, when plants were exposed to root herbivory with or without drought stress, *D. rapae* could differentiate between plants that were drought stressed but *A. colemani* could not. These findings suggest that root herbivory affected *A. colemani* more than *D. rapae.*

**Table 1:** Percentage time (mean minutes ± S.E.M.) spent by parasitoids in different treatment arms of an olfactometer compared with control arms.

Treatments	Parasitoid species (olfactometer)		Volatile emissions (RDA)		
	*Aphidius colemani*	*Diaeretiella rapae*	% variability explained by 1^st^ axis	r 1^st^ axis	F ratio
Control (−Rh) vs Drought stress (−Rh)	64.55±1.53 vs 21.92±1.01***	60.71±1.44 vs 23.46±0.88***	65.1	0.97	9.33 *
Control (−Rh) vs Drought stress (+Rh)	62.89±1.31 vs 22.70±1.07***	63.11±1.32 vs 22.07±1.29***	41.5	0.93	3.55 *
Drought stress (−Rh) vs Drought stress (+Rh)	47.73±1.44 vs 40.67±1.5**	42.46±1.47 vs 44.14±1.06	76.1	0.97	12.7
Control (+Rh) vs Drought stress (+Rh)	43.45±1.30 vs 40.12±1.38	55.78±2.49 vs 29.75±2.18***	19.8	0.77	1.48

Rh = root herbivore. Student t-Test was performed at 95% CI for comparison of means.

### Plant Volatile Emissions

**Figure 2:**
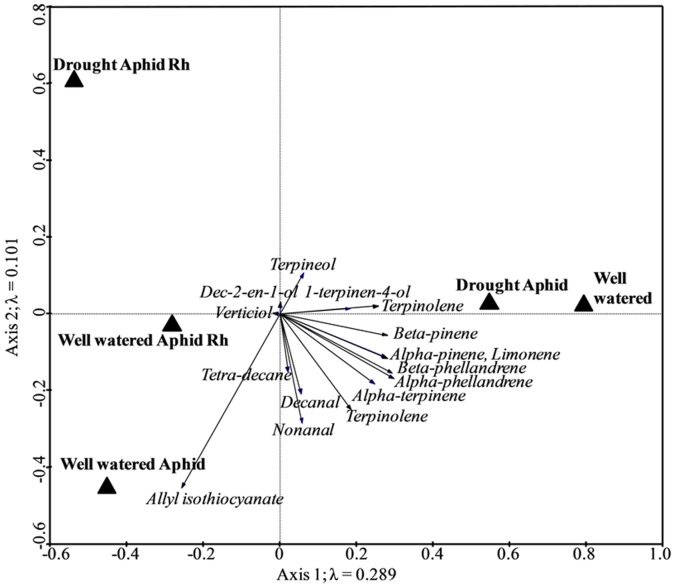
Constrained ordination diagram (redundancy analysis), showing effects of drought and well-watered treatments on VOC emissions from *Brassica oleracea* infested with *Brevicoryne brassicae* or root herbivore (Rh), both herbivores or neither.

Sixteen volatile compounds were identified: α-phellandrene, α-pinene, β-phellandrene, β-pinene, terpinolene, limonene, α-terpinene, terpineol, terpinolene, allyl isothiocyanate, nonanal, dec-2-en-1-ol, 1-terpinen-4-ol, decanal, tetradecane and verticiol (Figure 2), with significant treatment effects on the composition and concentration of VOCs (RDA Monte-Carlo permutation test; *F = *5.69; *P = *0.023). The first ordination axis (λ = 0.289) explains 63.5% of the variance in volatile emissions between the five treatments and separates uninfested, well-watered and aphid-infested drought stressed plants from the other treatments. The former are characterised by limonene, and α-pinene and β-phellandrene groups, and little or no allyl isothiocyanate. The second axis (λ = 0.101) explains an additional 22.3% of the variance, separating well-watered, aphid-infested plants from those with both herbivores, and is largely determined by the concentration of allyl isothiocyanate (Figure 2). Two-way treatment RDA comparisons showed significant differences between aphid-infested plants under well-watered vs. drought treatments, and between well-watered aphid-infested plants vs. drought stressed plants with both herbivores (Table 1).

**Figure 3:**
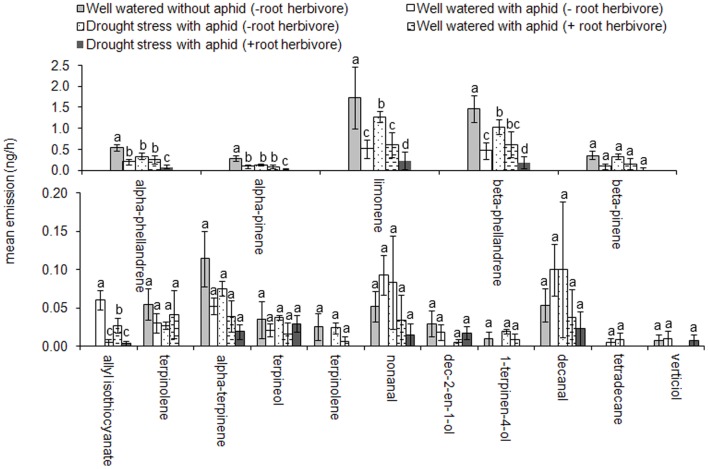
Individual VOC emissions (mean±S.E.M.) for 1) uninfested well-watered plants, 2) *Brevicoryne brassicae-*infested well-watered plants, 3) aphid infested drought stressed plants, 4) aphid and root herbivore infested well-watered plants, and 5) aphid and root herbivore infested drought stressed plants.

The GLM analyses for individual compounds showed significant differences in the emission of allyl isothiocyanate (*t* = 4.24; *P*<0.001), α -phellandrene (*t* = 3.75; *P*<0.05), β-phellandrene (*t = *3.40; *P*<0.05), α -pinene (*t* = 2.45; *P*<0.05) and limonene (*t = *2.76; *P*<0.05) under different treatments (Figure 3). The emission of α -phellandrene, β-phellandrene, α -pinene and limonene was reduced significantly on drought stressed plants with root herbivore (Tukey’s HSD, *P*<0.05). Allyl isothiocyanate was not detected from uninfested plants but was emitted from plants infested with *B. brassicae* (Tukey’s HSD, *P*<0.05) with the amount released being reduced when plants were also infested with *De. radicum* and further still when plants were drought stressed (Tukey’s HSD, *P*<0.05; Figure 3).

### Root Herbivore Performance

**Figure 4:**
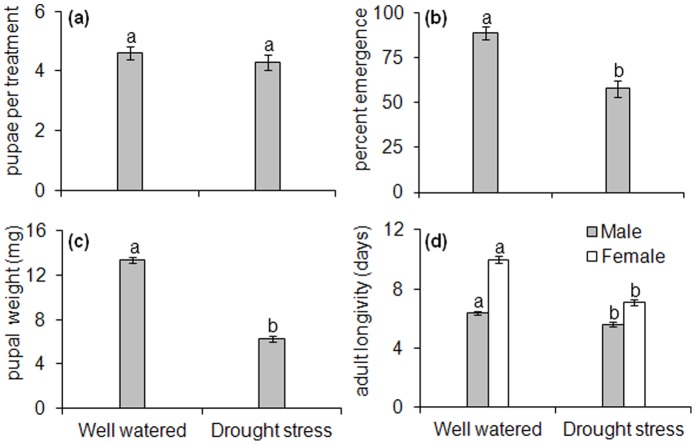
*Delia radicum* performance on well-watered (200 ml/pot/week; “Control”) and drought stressed plants (100 ml/pot/week; “Drought stress”). Means with different letters are significantly different (P<0.05): (a) pupae/treatment (b) percent emergence (c) pupal weight (d) adult longevity.

The number of larvae reaching pupation was not significantly different on control and drought stressed plants (*F*_1, 18_ = 0.7411; *P* = 0.4006, Figure 4a). Pupal weight (*F*_1, 58_ = 244.23; *P*<0.001, Figure 4b), percent adult emergence (*F*_1, 18_ = 25.963; *P*<0.001, Figure 4c), and adult longevity (*F*_1, 37_ = 15.52; *P*<0.001, Figure 4d) of *De*. *radicum* were significantly reduced by drought stress.

## Discussion

Here we found that biotic (*De*. *radicum* root herbivory) and abiotic (drought) stress influenced the preference and performance of the two aphid parasitoid species. Percentage parasitism and the proportion of females ovipositing were negatively affected by drought stress in the whole plant experiment, both of which indicate that female parasitoids assessed aphids feeding on drought stressed plants to be poor quality hosts [84], [85]. In addition, the presence of root herbivores reduced percentage parasitism of aphids on plants that were not drought stressed, and for one parasitoid species (*D. rapae*) reduced the proportion of ovipositing females. These results were similar to those of Soler et al. [74] on Brassica plants, where parasitoids preferred plants with undamaged roots on well watered plants. The effects of abiotic stress were not tested by Soler et al. [74]. Thus, parasitoids developed significantly better on foliar herbivores (hosts) that were feeding on undamaged plants (without root herbivory) [59]. This demonstratse that abiotic stress can alter the outcome of interactions between root herbivores and foliar herbivore parasitoids, as well as the foliar herbivores themselves [36], [61], [71]. Our first hypothesis, that high levels of drought stress and root herbivory combine to have a negative effect on parasitoid performance, is not completely supported by our results: drought stress was the dominant factor that reduced parasitism by *A*. *colemani* whereas either factor alone had the same negative effect as the combination of factors for *D*. *rapae*.

Parasitoid preference in olfactometer treatment comparisons was broadly similar to the percentage parasitism response found in the whole pot experiment; parasitoids preferred VOCs from well watered plants and percentage parasitism was higher on these too. Plant volatiles may therefore have played a role in parasitoid avoidance of drought-stressed plants as these are the only cues available to parasitoids in an olfactometer ([86]; see below for further discussion of volatiles). In contrast, the parasitoids’ choice in the olfactometer experiment between drought stressed plants with and without root herbivores, and plants with root herbivores that were either well watered (control) or under drought, did not correspond to the proportions of parasitised aphids in the whole pot experiment. When female parasitoids forage for hosts, it has been suggested that their behaviour can be categorized into five steps: habitat location, host location, host recognition, host acceptance and host suitability [87]–[89]. Olfactometer experiments only assess the first two stages of host location by parasitoids, and other cues such as non-volatile chemistry may become more important in the later stages. This demonstrates the importance of using a combination of approaches to assess host preference by parasitoids and rates of parasitism.

Parasitoid performance and preference depend on the ecology and physiology of both insect host and host plant. The plant mediated effects of root herbivores on aphid performance and abundance can have negative effects on aphid natural enemies [39], [60], [90]. Root herbivory-induced responses can influence the effectiveness of shoot-induced defence responses and can alter above-ground trophic interactions [14], [19]. In the present study, root herbivory influenced the parasitoid development as evidenced by reduced tibia length. Drought also reduced the emergence of adult parasitoids, though it had less of an effect on sex ratio or female tibia length than root herbivory. We have previously shown that both feeding by *De. radicum* and drought can increase concentrations of some foliar glucosinolate compounds, decrease foliar nitrogen concentration, and decrease leaf water content in this host plant [36], which may have reduced the quality of aphids as hosts for the developing parasitoids.

Plants often optimize their defensive investments according to abiotic growing conditions and herbivore pressure [91]. Drought stress in plants shifts the primary metabolism into the biosynthesis of the secondary metabolites [92], [93], thus water availability appears to be a regulatory factor for glucosinolate accumulation in Brassica plants [27], [36]. Similarly, the root herbivore (*De. radicum*) increases the glucosinolate concentration in Brassica plants [19]. This increase in glucosinolate concentration due to either drought or root herbivory can have a negative impact on the performance of foliar herbivores including aphids [36], [51], [55]–[58]. Studies have shown that glucosinolates have physicochemical properties that allow these endogenous compounds to be loaded and transported through phloem [94], [95] and have a negative impact on aphid performance [36]. This negative impact of root herbivory on foliar herbivore performance has been explained by the defence induction hypothesis, where foliar and root herbivores influence each other via induced changes in plant secondary compounds [6], [59]. Aphid fitness was also reduced due to the low amino acid concentration in the phloem and low leaf water contents of plants with root herbivory [59].

A few studies have demonstrated that the effects of root herbivores can be stronger for higher trophic levels than for the foliar herbivore itself [19], [60] and the effects can also influence hyperparasitism [19]. The developing larvae of parasitoids are highly vulnerable to the quality of their hosts. In the present study, the parasitoids avoided poor quality hosts (aphids), which developed under root herbivore attack. These parasitoids are under strong selection pressure to optimise their limited resources, as they develop in a single host. These results are in line with the optimal foraging theory, where carnivores select the most suitable host for maximum reward for them in term of their fitness [96].

The preference–performance hypothesis includes the prediction that selection pressure will favour phytophagous female invertebrates that oviposit on plants with high nutritional value, on which their offspring’s fitness is enhanced [97]–[99]. More recently this hypothesis has been extended to parasitoids, as increasing evidence shows that female parasitoids prefer to oviposit on hosts on which their offspring’s survival or fitness is increased [91], [100]. Since below-ground herbivory and drought stress directly affect plant quality, parasitoid success can be influenced by both factors [19], [41]. In the present study, there were some links between preference and performance, as both were maximised on well-watered control plants with no root herbivory. However, drought had a stronger effect on ‘preference’ in terms of decisions made on percentage parasitism and sex ratio by female parasitoids than root herbivory did. In contrast, performance in terms of female tibia length was reduced by root herbivory, but unaffected by drought stress for *A. colemani* and only slightly reduced for *D. rapae*. This suggests a mismatch between parasitoid preference (oviposition decisions) and performance under drought, and may relate to the strong effect of drought on the concentration of allyl isothiocyanate (discussed below). Under a future climate, drought may result in female parasitoids making oviposition decisions that are suboptimal for offspring.

Sequence of insect herbivore arrival is an important factor that may determine the outcome of plant mediated interactions between insect herbivores. In a recent meta analysis of above and below ground herbivore interactions, root herbivores impacted above ground herbivore only when both groups were introduced simultaneously, whereas above ground herbivores only affected root herbivore when arriving first [22]. Only a few studies have tested the outcome of an insect herbivore arriving before or after a second feeder on the performance of the latter. For example, the above ground herbivore (*Spodoptera frugiperda*) had a negative impact on the performance of a below ground herbivore (*Diabrotica virgifera*) when *S*. *frugiperda* arrived before *D. virgifera* [21]. However, interguild interactions between species pairs (i.e., chewer/sap-feeder) usually facilitate each other within and across domains [3]. Therefore, in the present study, aphids were introduced before the root herbivore and if aphids supressed plant defences this may have facilitated attack by the root herbivore. The potential mechanism behind this facilitation may be the eliciting of phytohormones that interfere with one another, thereby attenuating defences for the subsequent feeder [101]. Further studies should explore the mechanisms for interguild facilitation and also focus on the impact of sequence of insect herbivore arrival in multitrophic interactions.

Plants with root herbivores are often characterized as suboptimal food for foliar herbivores [59] but the foraging ability of an above-ground parasitoid can depend on root herbivore stage [74]. Parasitoids preferred hosts feeding on plants with final instars larvae of root herbivores (*De*. *radicum*) [74]. Parasitoids can also distinguish between infested and uninfested plants, and also discriminate between plants infested by different herbivore species [91]. This may be due to the modification of glucosinolate composition in root herbivore infested plants, which are precursors of volatile thiocyanates and isothiocyanates [102]. Further studies have shown that the plant VOC emissions can be determined by plant species as much as root glucosinolate profile and damage type [103], [104].

Plant VOCs emissions can vary both in quality and in quantity, depending on biotic and abiotic stress, and these changes can impact the attractiveness of the plants to natural enemies of the insect herbivores [68], [74], [105], [106]. Thus the quality and quantity of plant volatiles can change dramatically when plants are stressed [63], [74], [91], and the interactions between biotic and abiotic stress factors can have additive or opposing effects on plant volatile emissions [68]. Soler et al. [74] have demonstrated that the plant VOC emissions differed between undamaged plants and plants under attack by foliar or/and root herbivore. Plants with both foliar and root herbivores had volatile blends with lower concentration of attractants and higher concentration of sulfides compared with plants exposed to only foliar herbivore. This might be one of the main reasons that parasitoids in the present study avoided root damaged plants, as these plants had higher concentrations of toxic volatiles and lower levels of attractants (allyl isothiocyanate). Similarly, parasitoids avoided the drought stressed plants as they had low levels of attractants (see below). These results support our third hypothesis, that parasitoids will avoid aphid hosts feeding on plants under drought stress and root herbivory.

In the present study, no general pattern was observed for plant volatile emissions under different stresses as concentrations of some volatile compounds increased under drought and root herbivory, while others decreased. This may be due to the specific role of individual volatile compounds under single or multiple stresses [68]. The emission of general plant volatiles such as ß-phellandrene and limonene was greatest from uninfested plants compared with aphid infested plants. Emission of allyl isothiocyanate, a compound characteristic of brassicas which is known to be used as a host location cue by *D. rapae* [107], was highest in unstressed aphid infested plants. Drought stress caused a large reduction in the emission of allyl isothiocyanate almost to the level of plants with no aphid infestation in the current study, which may explain why such plants were less attractive to parasitoids. Root herbivory also reduced allyl isothiocyanate, but to a much lesser degree than drought. Epicuticular wax layers on leaves are known to increase on stressed plants, and this can reduce or inhibit volatile emission and may affect the foraging efficiency of *A*. *colemani* and *D*. *rapae* [70], [108]–[110]. Drought stress has been shown to reduce the rate of photosynthesis and increase stomatal closure, reducing the production of volatile compounds and their emission respectively [72]. Other studies [74], [75] have shown that parasitoid attraction can be significantly reduced under root herbivore attack. This suggests that both species used the same or similar infochemicals during foraging, as described by Steidle & Van Loon [111]. Our second hypothesis, that the VOC profile emitted by plants will be altered under drought stress and root herbivory treatments, is also supported by our results.

Studies have shown that sulphur containing compounds were emitted systemically by Brassica plants with roots infested by *De. radicum* [102], [104]. In the present study, we did not find any sulphur containing compound under root herbivore damage, which could be due to various reasons. These previous studies collected VOCs from roots of plants, while our focus was VOCs emitted from the above-ground parts of the plant, as this was the part the aphid parasitoids responded to. The quantity and quality of sulphur containing compounds differ among *Brassica* species and with the methods used to analyse plant VOCs from root herbivore infested plants [104]. One of these methods is the use of proton transfer reaction mass spectrometry (PTRMS) to analyse plant VOCs, which has high sensitivity as compared with traditional methods [103]. More sulphur containing compounds were detected using PTRMS as compared with standard electron impact GC-MS method that we used [102]. This may explain why we did not find any sulphur containing compounds in our analyses. It was also observed that the emission of sulphur containing compounds from plant roots may depend on *De. radicum* larvae themselves, bacteria and/or the plant material that can be present in their gut [102], [112]. The importance of transcription of genes and/or activation of enzymes has been reported for the production of sulfides in plants [113]. In addition to plant roots, soil microorganisms and plant pathogen may also contribute in the production of sulfide emissions, which are may be missing in our experiments [114], [115].

In the present study, drought stress had a negative impact on root herbivore performance, though it did not affect the proportion of larvae that survived to pupation, and so is unlikely to have changed the efficacy of the root herbivore treatment. The reduction in root herbivore performance is in agreement with previous studies [116]–[120], where drought stress influenced the performance and abundance of several root herbivores. We found drought stress had a negative impact on pupal weight, percent emergence and adult longevity of *De*. r*adicum*, and may be linked with poor food quality and/or limited food availability. The pupal weight of root herbivores on Brassica plants has been shown to have a positive correlation with root biomass [36], [121].

Our study shows for the first time that under drought stress, the strength of the interaction between root herbivory and parasitoids developing in above-ground herbivores insects can be changed. Despite a recognition of the need to include trophic interaction in climate change models [33], empirical evidence for the effects of climate change on such interactions is rare [32]. In our study, the response of our two parasitoid species to drought and root herbivory were broadly similar. This may make prediction of the effects of abiotic factors on interactions easier, though further studies are needed to confirm this. In addition, oviposition behaviour of female parasitoids under drought may not maximise performance of their offspring, leading to a potential reduction in parasitoid efficacy under drought. The influence of abiotic factors on indirect interactions between soil and above-ground food chains may play an important role in the structure and function of future terrestrial communities. Future studies should therefore focus on simultaneously testing the effects of multiple environmental factors, including drought, to determine how global climatic changes may impact the third and fourth trophic levels.

## Materials and Methods

### Ethics Statement

All work with insects was carried out according to the regulations of the Department of Environment, Food and Rural Affair, UK. This research work was also carried out according to the Policy on the Use of Animals in Research, and the Guidelines for Proper Scientific Conduct in Research, Central Secretariat, Imperial College London, UK. No protected species were used in this study.

### Study Species

*Brassica oleracea* L. var. *gemmifera* seeds were sown individually in pots (10 cm diameter) with John Innes No. 2 (Fargro Ltd, West Sussex, UK) compost and placed in a glasshouse with a minimum temperature of 20±2°C during the light period (16 h) and 14±2°C at dark (8 h). Overhead lighting (mercury halide and sodium) was supplied to ensure a minimum light intensity of 300 W/m^2^ during the light period. *Delia radicum* L. (Diptera: Anthomyiidae) pupae were obtained from the insect cultures maintained at HRI, University of Warwick, UK, and reared using the method described by Finch and Coaker [122]. *Aphidius colemani* Viereck (Hymenoptera: Aphidiidae) originating from commercial stocks of Just Green (Burnham-on-Crouch Essex, UK) and *Diaeretiella rapae* (McIntosh) (Hymenoptera: Aphidiidae) from Rothamsted Research (Harpenden, UK) were reared on separate cultures of *Myzus persicae* Sulzer (Sternorrhyncha: Aphididae) and *Brevicoryne brassicae* L. (Sternorrhyncha: Aphididae) respectively. *Myzus persicae* and *B. brassicae* were available from long-term culture established on 6-week-old *B. oleracea* plants. Both aphid species were sub-cultured fortnightly and transferred to fresh plants. The parasitoids were established on aphid species for at least two generations before use in the experiments [123] to minimize maternal host plant effects. Insect cultures were maintained at 20±2°C at 75% relative humidity under an LD 16∶8 h.

### Experimental Treatments

To assess the influence of drought stress on parasitoid performance and olfactory responses, *De. radicum* performance, and VOC emissions, six parallel series of plants (five replicates per treatments with two blocks) were grown in a greenhouse. Four weeks (after germination) old *B*. *oleracea* plants were moved to a control environment facility (20±2°C; 75% RH; LD 16∶8 h) where two water treatments were established. The quantity of water added per pot per week was 200 ml for the standard (control) water regime (well watered) and 100 ml for high drought stress as described previously. In a previous study [36], we had selected these quantities of water on the basis of a pilot experiment, where relative leaf water content of 11-week-old plants was used to quantify drought stress under root herbivore attack. The results of this study (data not shown) were used to select drought stress treatments for the main experiment. All the plants with a high density (5 larvae/plant) of *De*. *radicum* died at 50 ml water/plant/week and this treatment was discarded for the main experiment. The quantity of water added per pot per week for the main experiment was 200 ml for unstressed plants and 100 ml for high drought stress. These mentioned amounts of water were added once a week for each treatment [36]. After four weeks of drought stress treatments, three clip cages were fitted to the underside of 1^st^, 2^nd^ and 3^rd^ fully-developed leaves on each plant. Two separate batches of plants with five replicates per treatment were used for each aphid species. Since the performance of *G*_2_ of alate and apterae can differ [124], [125], the same form of aphid (apterae) was used. Clip cages and adult aphids were removed leaving one nymph per leaf on each treatment for four weeks. The sequence of arrival of herbivores on a host plant can affect the outcome of the interaction [21], [22] but the foraging ability of a parasitoid depends on the stage of the root herbivore (*De. radicum*) [74]. The parasitoids experiments required both aphids in large enough numbers and final instars larvae of *De*. *radicum* on each plant, therefore, aphids were introduced before the onset of root herbivory.

Two weeks after aphid treatments commenced, root herbivore treatments (five first instar root herbivore larvae vs a control without larvae) were introduced to the plants, carefully placing them with a camel hair brush onto the soil surface adjacent to the stem. Each plant was monitored for 30 min with a magnifying glass to ensure that all root herbivore larvae had entered into soil. *Delia radicum* were introduced to the plants after the aphids, as aphid performance was not assessed in the current study (but has been addressed previously; [36]). After four weeks (12-week-old plants) of aphid treatments, 300 aphids of each species were used to measure parasitoid performance, preference or plant volatile production. Extra aphids were removed from each plant. Plants infested with *B*. *brassicae* were used to assess the response of *D*. *rapae*; those infested with *M*. *persicae* were used with *A*. *colemani*. Plants infested with *B*. *brassicae* were used for the volatile entrainment work.

### Parasitoid Performance and Percentage Parasitism

Newly emerged females of each parasitoid species had been paired into a 2.5×8 cm glass tube and fed a single droplet of honey and a droplet of water daily [126]. As the percent parasitism is similar between aphid instars [127], the present studies were conducted on mixed instars, which were exposed to parasitoids (five replicates per treatment in two blocks). Five paired parasitoids (one pair per 60 aphids [128]) were released per replicate under ventilated bell cloches. Parasitoids were removed 24 h after release and remaining aphids were allowed to develop for 10–14 days for mummy formation [127]. Mummies were collected in individual gelatine capsules and percent parasitism, sex ratio (proportion of males), percent emergence, adult longevity, female hind tibiae length [89] and adult longevity recorded.

### Parasitoid Response to Plant Volatiles

The behavioural responses of *A*. *colemani* and *D*. *rapae* under each treatment were determined using a four-arm olfactometer [129]–[131] with a star-shaped arena with four regions (each 4 cm^2^) around a central orifice (2 cm^2^). Parasitoids could move freely within each region. Air was drawn in through the four orifices, the airflow for each quadrant being maintained at 100 ml/min using a vacuum pump (Capex 8C, Charles Austin Pumps Ltd, Byfleet, UK). Prior to the experiment a smoke test was used to confirm an equal airflow distribution.

Mummified aphids of *A*. *colemani* and *D*. *rape* were removed from their respective treatments and kept individually in vials (2 cm diameter×6 cm). On emergence females were mated within 24 h and fed on 50% aqueous solution of honey for 2 days. Naïve females (no previous oviposition experience) were used in all olfactometer tests [130]. Tests consisted of 12-week-old plants infested with 300 aphids under one of four treatments: well watered, drought, well watered with root herbivores; drought with root herbivores. Each choice bioassay (Table 1) consisted of a pairwise treatment comparisons (two plants per treatment), repeated five times with five female parasitoids in each repetition.

All bioassays were conducted at 20±2°C with 0.04 W/m^2^ (420–680 nm) light intensity [132]. A parasitoid was introduced into the central olfactometer chamber and left for 8 min. To control for directional bias in the chamber, the olfactometer was rotated 90° every 2 min [131]. The olfactometer was divided into five regions (four arms and centre) and the time spent in each region was recorded using Olfa software (F. Nazzi, Udine, Italy) [131] and converted to percent of total time. After every 10 parasitoids, the olfactometer was washed with Lipsol detergent (5% v/v; Bibby Sterilin Ltd., Staffordshire, UK), rinsed with 80% ethanol and air dried.

### Plant Volatiles

Air entrainment was used to trap VOCs from 12 week-old plants and GC-MS used to identify compounds [133]. The foliar part of each plant was enclosed in a 190×100 mm glass vessel. Two semicircular aluminium plates with a central hole to accommodate the stem were used to seal the bottom to exclude volatiles emitted from the soil and roots as much as possible. This glass vessel was closed at the top except for two ports (an inlet and an outlet). Air was pumped in through a charcoal filter with an airflow of 400 ml min^−1^. A Porapak Q (Alltech Associates Inc., Carnforth, UK) adsorbent glass tube (5 mm) with 50 mg Porapak Q was inserted into the outlet port and air was drawn through this tube at airflow rate of 300 ml min^−1^. This difference in airflow rate was used to create positive pressure to ensure that unfiltered air was not drawn into the vessel from outside. Twelve week-old plants were used for the collection of plant volatile compounds. GC-MS (HP5890) was equipped with a cold on-column injector, a flame ionization detector (FID), a non-polar HP-1 bonded-phase fused silica capillary column (50 m×0.32 mm i.d., film thickness 0.52 μ) and a polar DB-WAX column (30 m×0.32 mm i.d., film thickness 0.82 μ). The carrier gas was hydrogen. The oven temperature was kept at 30°C for two minutes and then programmed at 5°C per minute to 100°C and then temperature was maintained at 10°C per minute to 250°C. One µl of the concentrated air entrainment sample was used to inject inside the non-polar column.

The VOCs were collected from 1) uninfested well watered plants, 2) aphid (*B*. *brassicae*) infested well watered plants, 3) aphid infested drought stressed plants, 4) aphid and root herbivore infested well watered plants, and 5) aphid and root herbivore infested drought stressed plants.

### Root Herbivore Performance

Root herbivore performance (percent pupation and pupal dry weight [121]) was measured using the above treatments on separate batches of plants to assess the efficacy of the root herbivore treatment on drought and well-watered plants. Percent adult emergence and adult longevity were also assessed.

### Statistical Analysis

The effects of drought stress, root herbivory, parasitoid species and interactions between them on parasitoid performance were subjected to ANOVA. Data for parasitoid performance (percent parasitism, female tibia length, female adult longevity) and root herbivore performance (number of pupae, pupal weight, percent emergence, adult longevity) were log root transformed before analyses. Models were simplified by removing the blocks and any interactions that did not improve the statistical power [134], [135]. Within each parasitoid species, Posthoc Tukey HSD tests compared mean parasitoid performance. For olfactory responses, time spent in the treated region was converted to percent total time. Data was pooled between replicates and student’s t test was used to compare the mean after log or square root transformation if necessary. Plant VOCs were analysed using a constrained ordination method, redundancy analysis (RDA) in CANOCO version 4.5 for Windows [136]. RDA includes the option to test whether experimental treatments affect volatile composition through the use of Monte Carlo permutation tests [137]. RDA was conducted on all treatments and then repeated to compare the effects of the pairwise treatment combinations used in olfactometer experiments (section 2c) on plant volatile emissions. Treatment effects on the concentration of each VOC were also tested using GLM. With the exception of RDA, all statistical analyses were performed with R 2.14.1 [138].
